# 

**Published:** 1889-03

**Authors:** 


### SPECIAL OFFER.

As the present issue of the journal will reach a large number of new readers whom we desire to make constant readers, we will send the journal to such, for the remainder of the year, from March on, including the December number, for two dollars. Send in your check or P. O. order at once, as this offer only holds good until our next issue, April 15th.

